# Supplementary material for: Augmentation of frontoparietal gamma-band phase coupling enhances human altruistic behavior
Source: PLoS Biol. 2026 Feb 10;24(2):e3003602. doi: 10.1371/journal.pbio.3003602 (PMC12890155; doi:10.1371/journal.pbio.3003602)
Supplement: S1 Table — (DOCX) [file pbio.3003602.s001.docx]

**S1 Table. Logistic mixed-effects model results of choice data for DIS context.**

|  | Model 1 | | Model 2 | | Model 3 | | Model 4 | |
| --- | --- | --- | --- | --- | --- | --- | --- | --- |
| Fixed effects | $\beta$  (95% CI) | p-value | $\beta$  (95% CI) | p-value | $\beta$  (95% CI) | p-value | $\beta$  (95% CI) | p-value |
| Intercept | -2.70***  (-3.11 – -2.30) | < 0.001 | -2.71***  (-3.12– -2.30) | < 0.001 | -2.73***  (-3.14 – -2.32) | < 0.001 | -2.69***  (-3.10 – -2.28) | < 0.001 |
| Gamma (G) | 0.18*  (-0.01 – 0.37) | 0.035 | 0.19*  (-0.01 – 0.38) | 0.029 | 0.21 *  (-0.001 – 0.42) | 0.025 | 0.17*  (-0.03 – 0.37) | 0.046 |
| Sham  (S) | -0.01  (-0.21 – 0.19) | 0.929 | - | - | 0.04  (-0.20 – 0.27) | 0.754 | - | - |
| Alpha  (A) | - | - | 0.01  (-0.19 – 0.21) | 0.929 | - | - | -0.04  (-0.27 – 0.20) | 0.754 |
| Discomfort rating | - | - | - | - | 0.08  (-0.13 – 0.29) | 0.469 | 0.08  (-0.13 – 0.29) | 0.469 |
| Intensity | - | - | - | - | 0.01  (-0.37 – 0.40) | 0.951 | 0.01  (-0.37 – 0.40) | 0.951 |
| Conditional R^2^ | 0.32 | | 0.32 | | 0.32 | | 0.32 | |
| LL | -2078 | | -2078 | | -2077 | | -2077 | |
| BIC | 4191 | | 4191 | | 4208 | | 4208 | |

Tests for the effect of “Gamma” entrainment were implemented with one-tailed statistical tests. Discomfort rating, participants rated their discomfort due to the stimulation after each entrainment/stimulation block. Intensity, each participant was stimulated with an electric current intensity on his/her tolerance level for the stimulation currents tested before the experiment runs. Gamma (G): gamma entrainment; Sham (S): sham stimulation; Alpha (A): alpha entrainment; LL: log-likelihood; BIC: Bayesian Information Criterion. ***, *p* < 0.001; **, *p* < 0.01; *, *p* < 0.05.
