# Supplementary material for: Augmentation of frontoparietal gamma-band phase coupling enhances human altruistic behavior
Source: PLoS Biol. 2026 Feb 10;24(2):e3003602. doi: 10.1371/journal.pbio.3003602 (PMC12890155; doi:10.1371/journal.pbio.3003602)
Supplement: S2 Table — (DOCX) [file pbio.3003602.s002.docx]

**S2 Table. Logistic mixed-effects model results of choice data for ADV context.**

|  | Model 1 | | Model 2 | | Model 3 | | Model 4 | |
| --- | --- | --- | --- | --- | --- | --- | --- | --- |
| Fixed effects | $\beta$  (95% CI) | p-value | $\beta$  (95% CI) | p-value | $\beta$  (95% CI) | p-value | $\beta$  (95% CI) | p-value |
| Intercept | -2.30***  (-2.87 – -1.73) | < 0.001 | -2.23***  (-2.80– -1.66) | < 0.001 | -2.29***  (-2.86 – -1.72) | < 0.001 | -2.23***  (-2.80 – -1.67) | < 0.001 |
| Gamma (G) | 0.12  (-0.05 – 0.30) | 0.075 | 0.05  (0.12 – 0.22) | 0.28 | 0.12  (-0.08 – 0.31) | 0.118 | 0.06  (-0.12 – 0.23) | 0.265 |
| Sham  (S) | 0.07  (-0.10 – 0.25) | 0.399 | - | - | 0.06  (-0.15 – 0.27) | 0.583 | - | - |
| Alpha  (A) | - | - | -0.07  (-0.25 – 0.10) | 0.399 | - | - | -0.06  (-0.27 – 0.15) | 0.583 |
| Discomfort rating | - | - | - | - | -0.03  (-0.27 – 0.22) | 0.827 | -0.03  (-0.27 – 0.22) | 0.827 |
| Intensity | - | - | - | - | 0.19  (-0.37 – 0.74) | 0.505 | 0.19  (-0.37 – 0.74) | 0.505 |
| Conditional R^2^ | 0.51 | | 0.51 | | 0.51 | | 0.51 | |
| LL | -2495 | | -2495 | | -2495 | | -2495 | |
| BIC | 5025 | | 5025 | | 5042 | | 5042 | |

Tests for the effect of “Gamma” entrainment were implemented with one-tailed statistical tests. Discomfort rating, participants rated their discomfort due to the stimulation after each entrainment/stimulation block. Intensity, each participant was stimulated with an electric current intensity on his/her tolerance level for the stimulation currents tested before the experiment runs. Gamma (G): gamma entrainment; Sham (S): sham stimulation; Alpha (A): alpha entrainment; LL: log-likelihood; BIC: Bayesian Information Criterion. ***, *p* < 0.001; **, *p* < 0.01; *, *p* < 0.05.
