# Supplementary material for: Augmentation of frontoparietal gamma-band phase coupling enhances human altruistic behavior
Source: PLoS Biol. 2026 Feb 10;24(2):e3003602. doi: 10.1371/journal.pbio.3003602 (PMC12890155; doi:10.1371/journal.pbio.3003602)
Supplement: S3 Table — (DOCX) [file pbio.3003602.s003.docx]

**S3 Table. Model comparison results.**

| Model | Parameters | BIC (mean ± SE) | $\Delta BIC$ | BF |
| --- | --- | --- | --- | --- |
| M1 | $\omega_{\left( c, s \right)}$, $\lambda_{\left( s \right)}$ | 202 ± 14 | 0 | 1 |
| M2 | $\omega_{\left( c, s \right)}$, $\lambda_{\left( c, s \right)}$ | 218 ± 15 | 16 | 2.98 × 10^3^ |
| M3 | $\omega_{\left( c, s \right)}, \mu_{\left( c,s \right)},$ $\lambda_{\left( s \right)}$ | 236 ± 14 | 34 | 2.42 × 10^7^ |
| M4 (SSM) | $\omega_{\left( c, s \right)}, a_{\left( c,s \right)},z_{\left( c,s \right)},$ $k_{\left( c, s \right)}, \tau_{\left( c,s \right)},\gamma_{\left( c,s \right)}$ | 3019 ± 52 | 2817 | Inf |

BIC, Bayesian Information Criterion; BF, Bayes factor. SSM, sequential sampling model. $\omega_{\left( c, s \right)}$, relative weight on others’ payoffs; $\lambda_{\left( c, s \right)}$, inverse temperature parameter;$\mu_{\left( c,s \right)}$, relative weight on efficiency; $a_{\left( c,s \right)}$, decision threshold; $z_{\left( c,s \right)}$, starting point bias; $k_{\left( c, s \right)}$, drift rate modulator; $\tau_{\left( c,s \right)}$, non-decision time; $\gamma_{\left( c,s \right)}$, leak strength; c for conditions (c = DIS & Gamma, DIS & Sham, DIS & Alpha for different stimulation conditions in disadvantageous inequality context, and c = ADV & Gamma, ADV & Sham, ADV & Alpha for different stimulation conditions in advantageous inequality context), s for participants (s = 1, ..., N_participants_). SE, standard error.
