# Supplementary material for: Augmentation of frontoparietal gamma-band phase coupling enhances human altruistic behavior
Source: PLoS Biol. 2026 Feb 10;24(2):e3003602. doi: 10.1371/journal.pbio.3003602 (PMC12890155; doi:10.1371/journal.pbio.3003602)
Supplement: S4 Table — (DOCX) [file pbio.3003602.s004.docx]

**S4 Table. Logistic mixed-effects model results of choice data for ambiguous trials.**

|  | Model 1 | | Model 2 | | Model 3 | | Model 4 | |
| --- | --- | --- | --- | --- | --- | --- | --- | --- |
| Fixed effects | $\beta$ (95% CI) | p-value | $\beta$ (95% CI) | p-value | $\beta$ (95% CI) | p-value | $\beta$ (95% CI) | p-value |
| Intercept | 0.24***  (0.14 – 0.34) | < 0.001 | 0.23***  (0.13 – 0.33) | < 0.001 | 0.24***  (0.14 – 0.34) | < 0.001 | 0.23***  (0.13 – 0.33) | < 0.001 |
| Inequality context (C) | -0.42 ***  (-0.55 – -0.28) | < 0.001 | -0.38***  (-0.52 – -0.25) | < 0.001 | -0.42 ***  (-0.55 – -0.28) | < 0.001 | -0.38***  (-0.52 – -0.25) | < 0.001 |
| Gamma (G) | -0.03  (-0.17 – 0.10) | 0.635 | -0.02  (-0.16 – 0.12) | 0.748 | -0.04  (-0.17 – 0.10) | 0.618 | -0.02  (-0.16 – 0.12) | 0.759 |
| Sham (S) | -0.01  (-0.15 – 0.13) | 0.879 | - | - | -0.01  (-0.15 – 0.13) | 0.849 | - | - |
| Alpha (A) | - | - | 0.01  (-0.13 – 0.15) | 0.879 | - | - | 0.01  (-0.13 – 0.15) | 0.849 |
| G*C | 0.07  (-0.12 – 0.26) | 0.486 | 0.04  (-0.15 – 0.23) | 0.700 | 0.07  (-0.12 – 0.26) | 0.485 | 0.04  (-0.15 – 0.23) | 0.699 |
| S*C | 0.03  (-0.16 – 0.22) | 0.755 | - | - | 0.03  (-0.16 – 0.22) | 0.755 | - | - |
| A*C | - | - | -0.03  (-0.22 – 0.16) | 0.755 | - | - | -0.03  (-0.22 – 0.16) | 0.755 |
| Discomfort rating | - | - | - | - | -0.005  (-0.05 – 0.04) | 0.819 | -0.005  (-0.05 – 0.04) | 0.819 |
| Intensity | - | - | - | - | 0.02  (-0.02 – 0.06) | 0.289 | 0.02  (-0.02 – 0.06) | 0.289 |
| LL | -6898 | | -6898 | | -6897 | | -6897 | |
| BIC | 13860 | | 13860 | | 13878 | | 13878 | |

Analyzing only the ambiguous trials, only the main effect of inequality context (C) was significant. The four models were constructed in the same way as Tables 2 and 3 reported in the main text. One-tailed tests for the effects of “Inequality context” and “Gamma” entrainment, but two-tailed statistics for the other effects with undirected hypotheses. Discomfort rating, participants rated their discomfort due to the stimulation after each entrainment/stimulation block. Intensity, each participant was stimulated with an electric current intensity on his/her tolerance level for the stimulation currents tested before the experiment runs. Gamma (G): gamma entrainment; Sham (S): sham stimulation; Alpha (A): alpha entrainment; LL: log-likelihood; BIC: Bayesian Information Criterion. ***, *p* < 0.001; **, *p* < 0.01; *, *p* < 0.05.
