# Supplementary figures and images for: Augmentation of frontoparietal gamma-band phase coupling enhances human altruistic behavior
Source: PLoS Biol. 2026 Feb 10;24(2):e3003602. doi: 10.1371/journal.pbio.3003602 (PMC12890155; doi:10.1371/journal.pbio.3003602)

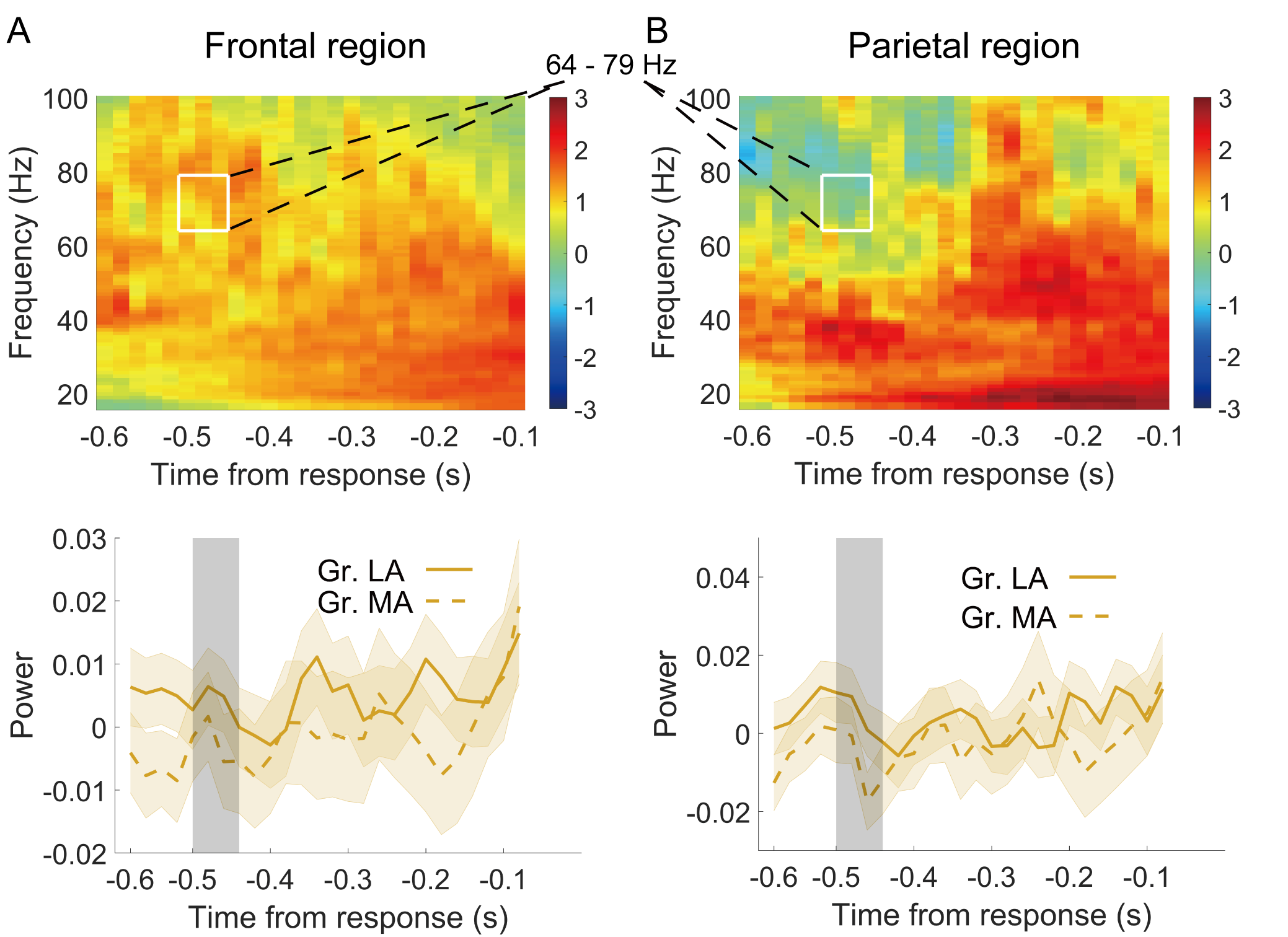

Supplement: S1 Fig — (A and B top panel) Heatmap showing T-statistics for the differences between the more altruistic (Gr. MA) minus less altruistic (Gr. LA) group in local power of frontal cluster (A) shown in Fig 5A of our original EEG paper and parietal cluster (B) shown in Fig 2C of our original EEG paper (https://elifesciences.org/articles/80667#fig5). No significant cluster survived cluster correction for multiple comparisons at p < 0.05. Moreover, no significant effect was identified in the gamma-band frequency range (~64–79 Hz) at the time window of ~520–460 ms before response (highlighted in white box) either in the frontal or in the parietal region. In contrast, the averaged dWPLI between frontal and parietal regions in this time-frequency window (~64–79 Hz and ~520–460 ms before response) correlated with individuals’ altruistic preferences in our EEG paper. (A and B bottom panel) Temporal dynamics of the average power change relative to the baseline in the ~ 64–79 Hz frequency range. Gray shaded area indicates the duration of ~520–460 ms before response. Colored shaded areas indicate ±1 SEMs. Gr., group. Significant clusters surviving the cluster correction for multiple comparisons at p < 0.05 are reported, N(LA) = 19, N(MA) = 19. Gr. MA, more altruistic group; Gr. LA, less altruistic group. (TIF) [file pbio.3003602.s005.tif]

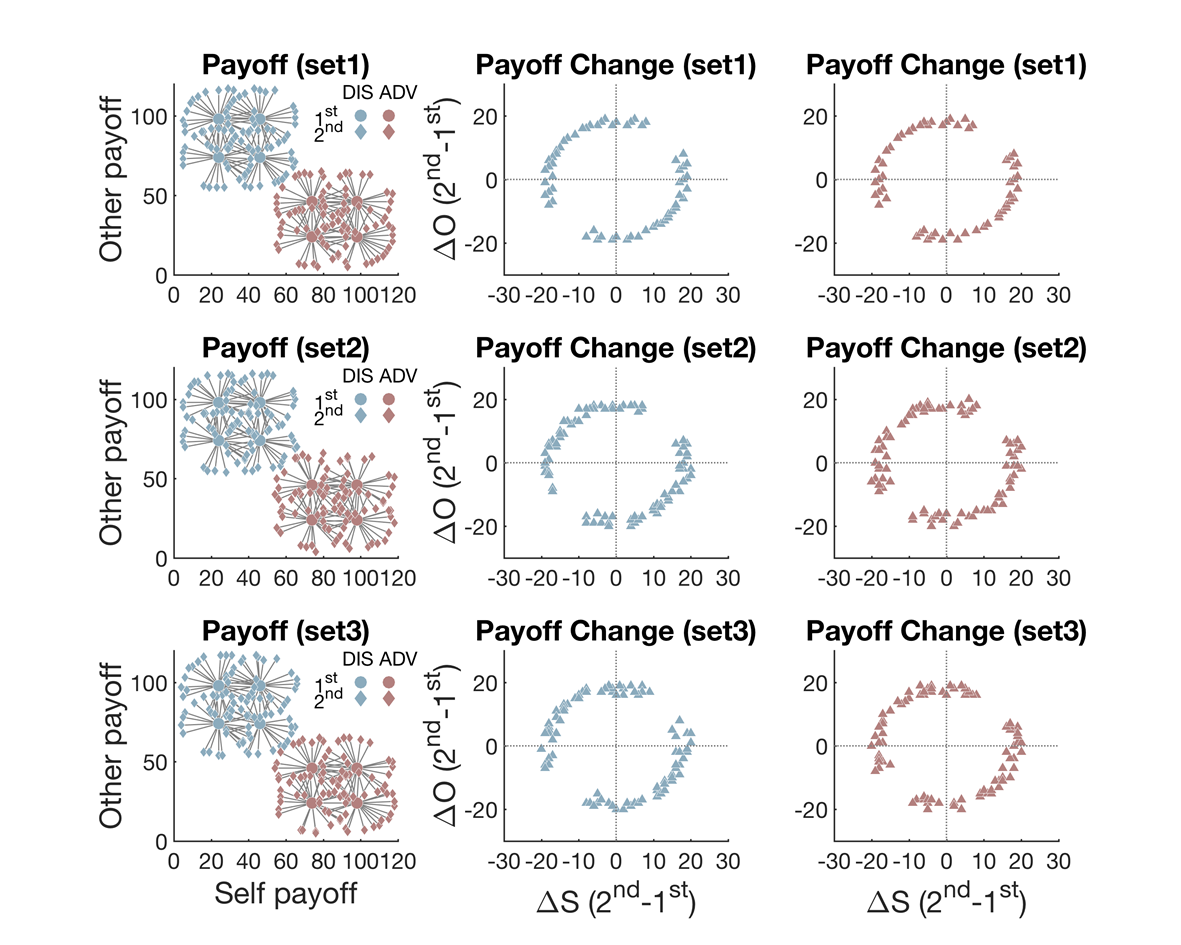

Supplement: S2 Fig — We included two inequality contexts: disadvantageous inequality (DIS) and advantageous inequality (ADV). In the left panel, each dot represents one allocation option and each gray line represents one pair of options that was presented to participants. Blue dots are options in DIS and pink dots are options in ADV. Dots in the center of the circle are the 1st options and diamond dots are the 2nd options. Middle and right panels show the distributions of the self-/other-payoff changes between the 2nd and the 1st option (ΔS and ΔO) in DIS and ADV, respectively. These three sets of payoff matrices (top, middle, and bottom panel) have the same reference options and similar distributions of alternative options. By having such payoff matrices of all trials, we matched self-/other-payoff differences and the resulting absolute levels of inequality across both contexts and also across the 2nd and the 1st options. This allowed us to compare choices as well as neural processing of different choice features (self- and other-payoff, inequality), between the two contexts. (TIF) [file pbio.3003602.s006.tif]

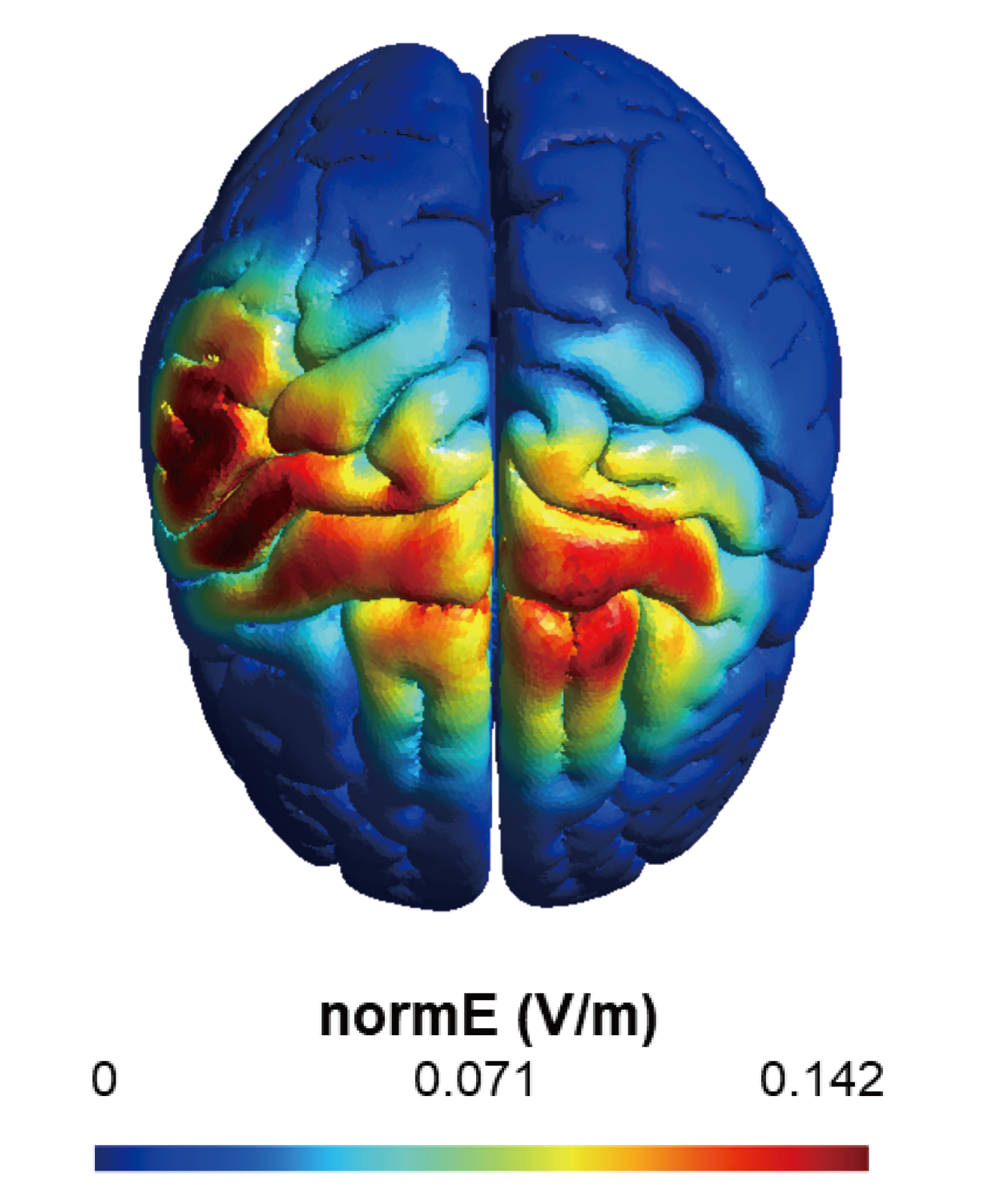

Supplement: S3 Fig — Two sets of small electrodes (3 × 1 HD electrode montage) were mounted over frontal and parietal regions which were located based on our previous EEG study [18]. The normalized simulated electric field distribution showed that the targeted parietal and frontal areas under the two sets of tACS electrodes are affected by the stimulation with a poor spatial focality for asynchronous entrainment. (TIF) [file pbio.3003602.s007.tif]

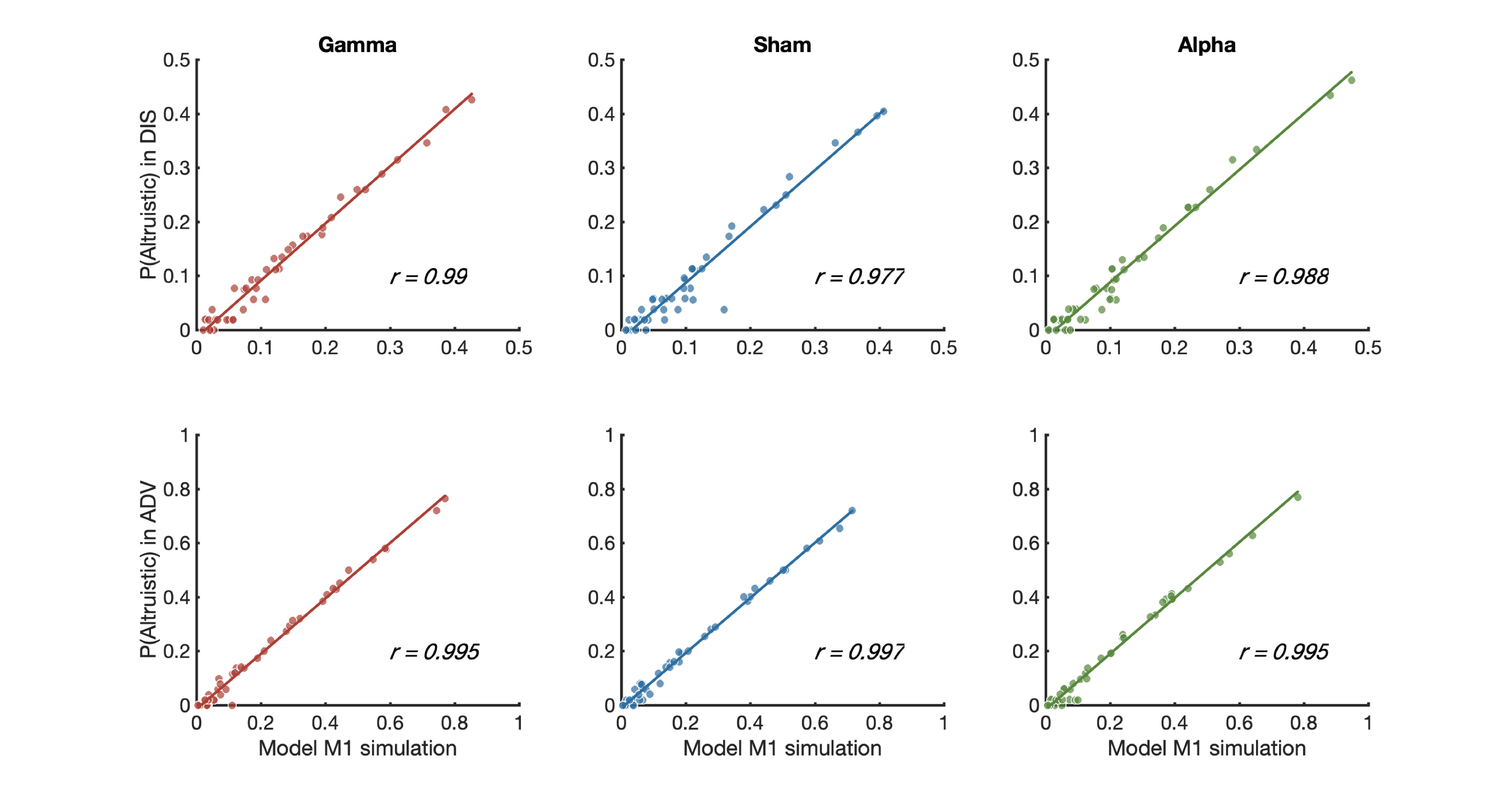

Supplement: S4 Fig — Correlations of the probability of altruistic choice between participants’ true responses (y axis) and model simulation responses (x axis) based on the winning model, for DIS context (top panel) and for ADV context (bottom panel). Model simulation data are highly correlated with observed true data across all inequality contexts and stimulation types. (TIF) [file pbio.3003602.s008.tif]

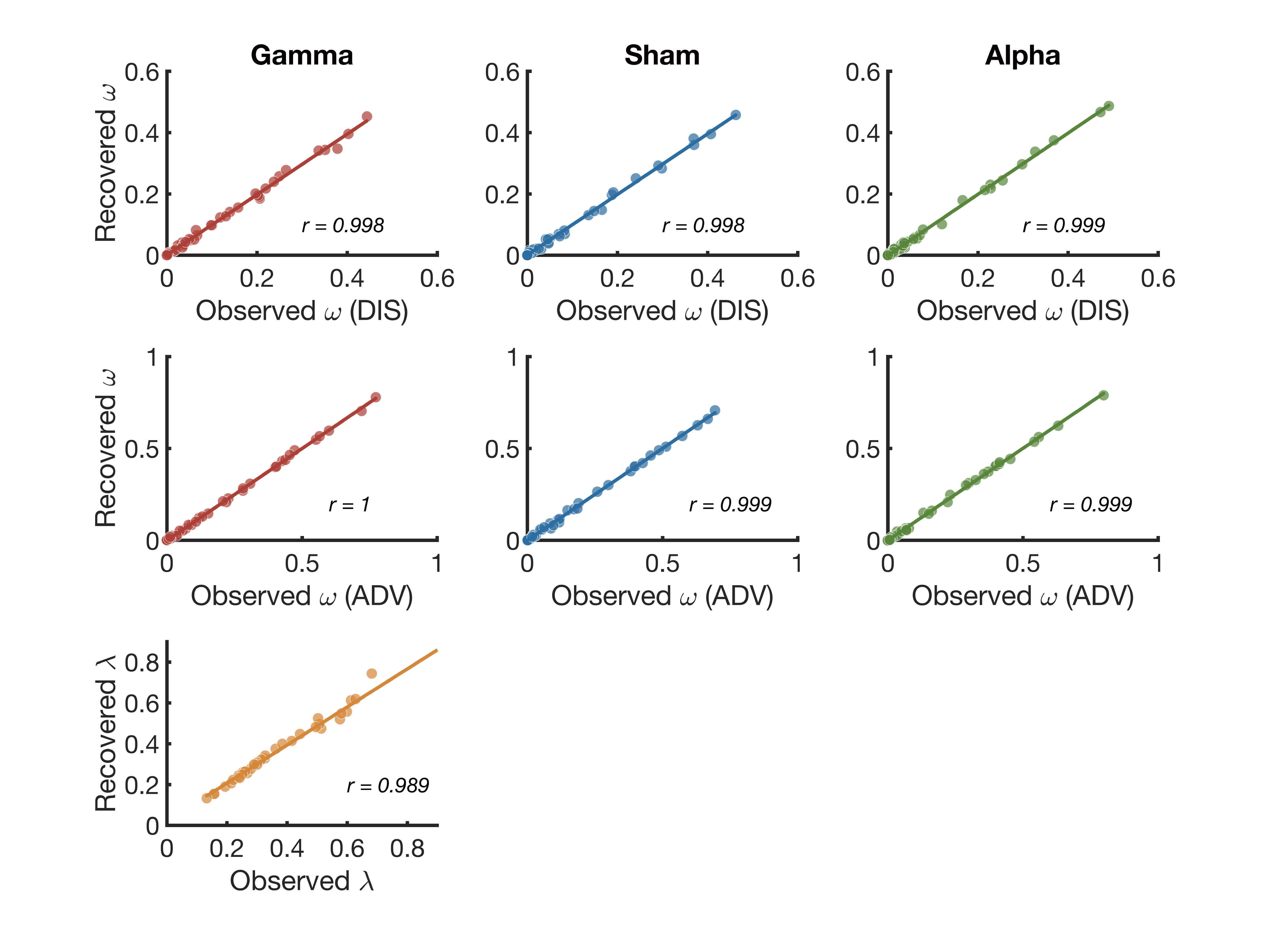

Supplement: S5 Fig — Observed parameters (values fitted to participants’ choice data in the winning model M1) are used to simulate choices, and these choices are used to recover the observed parameters. We repeated this procedure 100 times to obtain the average value of each recovered parameter and correlated the averaged recovered parameters with the observed parameters (which were used to generate the choices). Each dot plots the averaged recovered parameter from the simulated choices against the observed parameter (generating parameter), and the colored lines represent the regression fits of the observed parameters on the recovered parameters. (TIF) [file pbio.3003602.s009.tif]

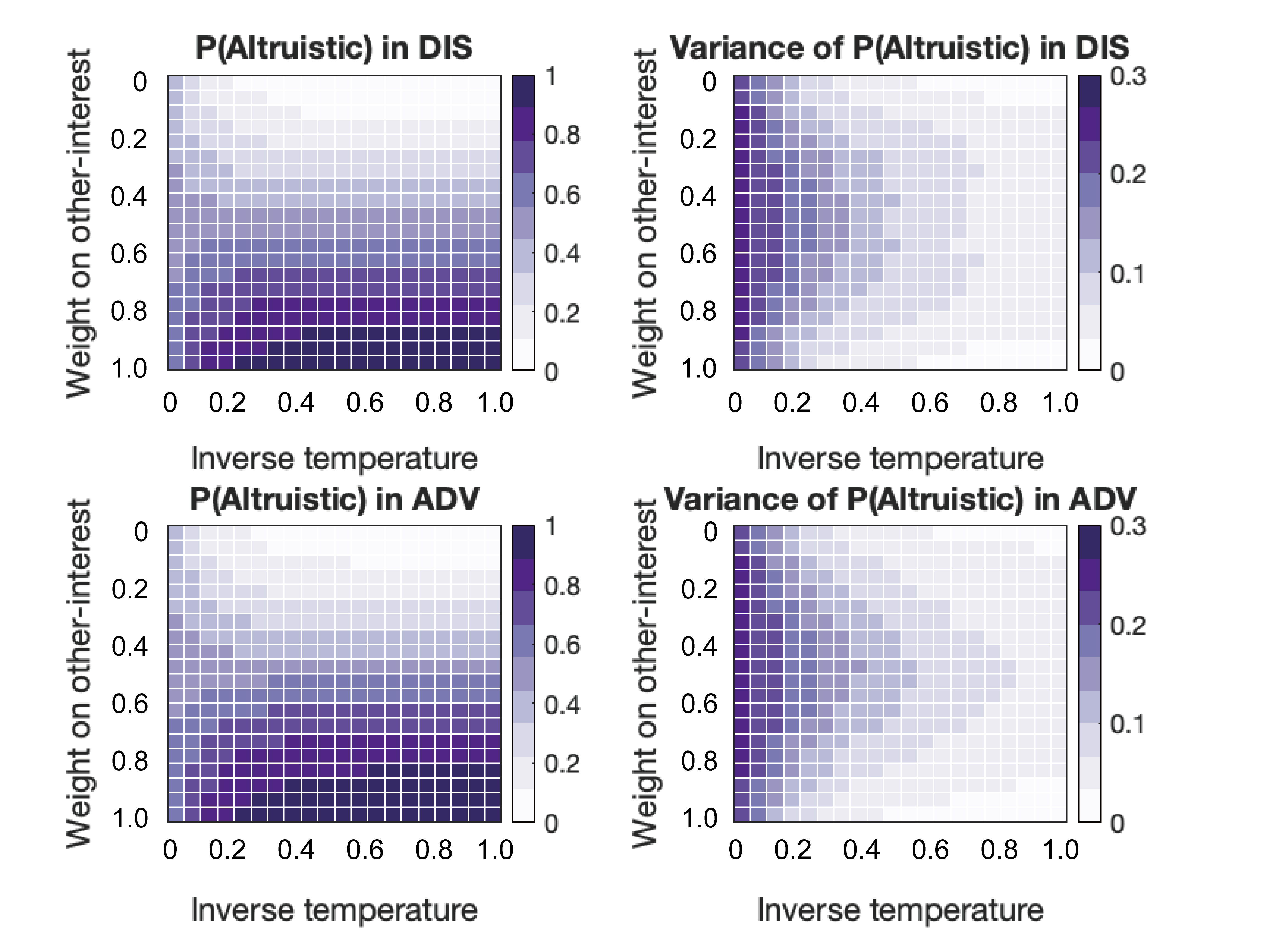

Supplement: S6 Fig — The overall probability of altruistic choice mainly increases with the weight on other-interest for both contexts (left panel), while the variance of altruistic choice will mainly decrease with the inverse temperature for both contexts (right panel), suggesting that the two parameters in the softmax function model used here is good to capture the effects of weight on other-interest and the decision precision on altruistic choice. (TIF) [file pbio.3003602.s010.tif]

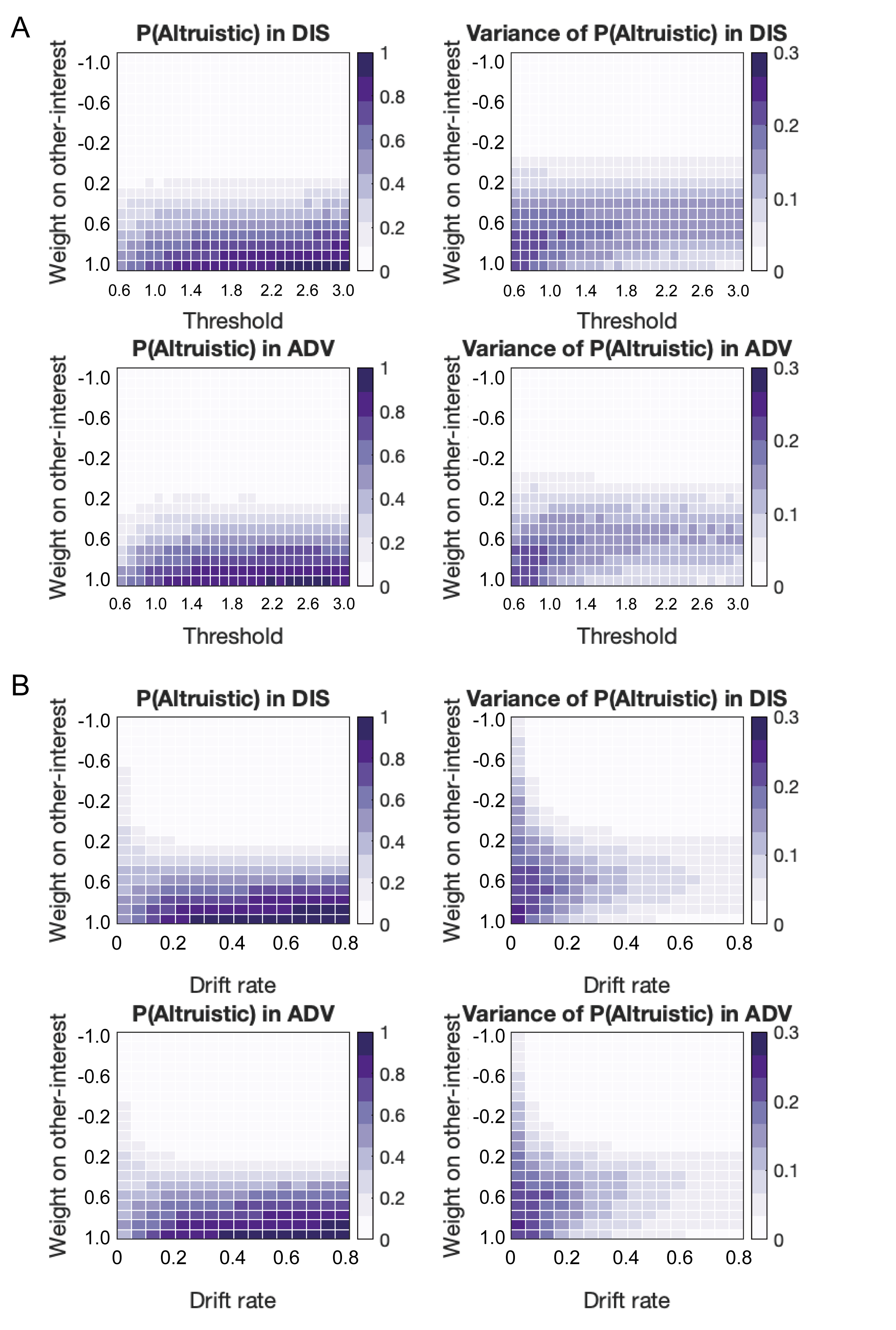

Supplement: S7 Fig — The probability of altruistic choice increasing with weight on other-interest (A and B left panel) while variance of altruistic choice increasing with weight on other-interest and decreasing with both decision threshold (A right panel, upper panel for DIS and lower panel for ADV) and drift rate (B right panel, upper panel for DIS and lower panel for ADV). (TIF) [file pbio.3003602.s011.tif]
