# Supplementary material for: Augmentation of frontoparietal gamma-band phase coupling enhances human altruistic behavior
Source: PLoS Biol. 2026 Feb 10;24(2):e3003602. doi: 10.1371/journal.pbio.3003602 (PMC12890155; doi:10.1371/journal.pbio.3003602)
Supplement: Attachment — Submitted filename: Response to comments_plos biology_v6.docx [file pbio.3003602.s014.docx]

| Zurich, November 1, 2025 | |
| --- | --- |
|  | |

Dear Dr Hart,

We thank the reviewers for their careful reading of our manuscript and their constructive suggestions. We have revised the manuscript accordingly. Below, we provide a point-by-point response to each comment.

**Reviewer #1:**

*This study investigates the possibility of increasing altruistic behaviour by delivering transcranial alternating current stimulation (tACS) over frontal and parietal regions, which putatively increases fronto-parietal coherence. The authors found that applying tACS over these regions increased altruism, particularly during disadvantageous inequality. The manuscript is clear and well-written; the study is well-thought and methodologically sound. The computational models are well formulated, stable and robust across the parameter ranges. I do, however, have a few comments and suggestions that I detail below.*

*Major points:*

*-Introduction: the authors have written a clear and eloquent introduction, including the relevant literature that nicely builds up towards the experimental questions. I do, however, miss the hypothesis / predictions of the results at the end for the introduction, e.g. after the research questions.*

**Response:**

Thanks for this suggestion! We now concisely state our hypotheses at the end of the Introduction. (*see below*). We now explicitly describe our hypotheses that (1) gamma-band fronto-parietal synchronization will enhance altruistic preferences, and this effect will be specific to gamma- rather than other frequency band (i.e., alpha-frequency) stimulation, and (2) the enhancement will be most pronounced in the disadvantageous inequality context.

New text on *p. 8*:

“Based on prior evidence linking gamma-band oscillations to value integration and evidence accumulation, we expected that enhancing gamma-band synchronization between frontal and parietal regions would promote altruism, whereas stimulation in other frequency bands (i.e., alpha band) would not generate such effects. Moreover, we anticipated that this facilitation would be most pronounced in contexts of disadvantageous inequality, since the strength of frontoparietal phase-coupling was correlated with altruistic preferences mainly during disadvantageous inequality.”

-*Increasing interregional synchronisation with tACS: the authors describe a clear tACS procedure in which they putatively entrain interregional coherence between the frontal and parietal areas at specific frequency bands - i.e. gamma and alpha. Although, it is likely that a tACS protocol tuned at these specific frequency bands lead to an entrainment of such frequency bands during the task, a different (alternative) impact of the tACS on EEG oscillatory synchrony cannot be ruled out. This is, it is possible that tACS' effect on interregional communication between the areas targeted might not have had the expected effect. It would have been beneficial to directly measure the effects of tACS on interregional coherence with EEG recordings, at least for a subset of the participants. The examination of the EEG recordings before vs during (or after) the tACS intervention would have revealed the direct (causal) impact of the tACS in fronto-parietal coherence, significantly*

*strengthening the interpretation of the results. It would be advisable that the authors are able to show in a subset of participants the direct effect of frequency-specific tACS on EEG coherence between the frontal and parietal regions. At the very least, the authors should acknowledge this limitation in the discussion and interpretation of the results.*

**Response:**

We thank the reviewer for this important comment and fully agree that simultaneous tACS-EEG would provide the most direct evidence for frequency-specific entrainment. However, implementing such a setup was not feasible in the current study. As suggested by the reviewer, we now explicitly acknowledge this limitation, emphasizing that our conclusions are based on behavioral effects rather than direct physiological measures. We also state that an important direction for future work will be to combine tACS with EEG to directly validate the neural impact of the stimulation protocol.

We now discuss this issue in the Discussion section of the revised manuscript (*p. 23*): “A limitation of the present study is that we did not directly record neural activity during the stimulation procedure. While our tACS protocol was designed to modulate fronto-parietal coherence at specific frequency bands (Polanía et al., 2015), we cannot provide direct neural measures of the intended entrainment. Without concurrent EEG recordings, it remains theoretically possible that stimulation may have affected behavior also through mechanisms other than frequency-specific synchronization between the targeted regions. While technically challenging (Gebodh et al., 2019; Mancini et al., 2015), future studies combining tACS with EEG may validate the frequency- and network-specific impact of the stimulation, to more directly link moment-to-moment changes in fronto-parietal gamma-band coherence to altruistic decision-making.”

*Minor points:*

*-Abstract: the abstract mention something about EGG findings. I wonder if the authors mean EEG findings.*

**Response:**

We are sorry for this typo, it is now corrected (*p. 2*).

*-Introduction: the authors nicely explain the concept of inter-regional coherence and how this can be modulated with different NIBS protocols. I think the introduction would benefit from including other NIBS protocols that have been used to selectively manipulate intra-areal coherence testing the Communication Through Coherence Hypothesis - for example: Trajkovic, J., Romei, V., Rushworth, M. F., & Sel, A. (2023). Changing connectivity between premotor and motor cortex changes inter-areal communication in the human brain. Progress in Neurobiology, 228, 102487.*

**Response:**

Thanks for this helpful suggestion! We now cite Trajkovic et al. (2023) and briefly discuss other NIBS protocols that have been used to manipulate inter-areal coherence and test the communication-through-coherence hypothesis. This addition highlights the broader feasibility of using NIBS to study neural communication. Building on these approaches, we chose to employ high-definition tACS in the current study, as it allows for frequency-specific modulation of long-range fronto-parietal synchronization and thus extends previous work by directly testing its causal role in altruistic decision-making.

We now clarify this issue in the Introduction section of the revised manuscript (*p. 6*): “Beyond correlative evidence for the general functional importance of inter-regional connectivity, recent advances in non-invasive brain stimulation (NIBS) protocols have provided causal tests of the “communication through coherence” hypothesis (Fries, 2015). For instance, paired stimulation approaches using TMS stimulators have been shown to selectively manipulate inter-areal coherence between premotor and motor cortices, thereby altering inter-regional communication (Trajkovic et al., 2023). These studies highlight the feasibility of experimentally modulating neural coherence to probe its causal role in cognition and behavior. Consistent with the general rationale underlying this line of work, we here employed high-definition tACS to target fronto-parietal synchronization, to directly examine whether exogenous modulation of frequency-specific long-range coherence can shape altruistic decision-making.”

**Reviewer #2:**

*This paper investigates the causal role of gamma rhythms within the frontoparietal network in encoding other-related prosocial acts - altruistic behaviors. The study uses an elegant experimental design that effectively measures altruistic behavior, building upon prior EEG findings that linked frontoparietal gamma frequencies with altruistic tendencies. By using tDCS stimulation to modulate gamma rhythms in the frontoparietal network, the authors demonstrate a causal involvement of these frequencies in altruistic acts in the context of disadvantageous inequity. The precise mechanisms underlying altruistic strategies were detected by applying computational modeling, demonstrating the causal role of gamma rhythms in assigning weight to other-regarding concerns during the decision-making process. This work is of significant interest for scientific research in cognitive neuroscience and neuroeconomics due to its novel findings on the causal manipulation of altruistic behavior. However, the current work is not yet ready for publication and requires substantial revisions to strengthen its findings and interpretations.*

*Major Revisions*

*The current behavioural modeling approach lacks sufficient clarity and must be simplified:*

*(1) Table 1 and the description suggest that tDCS conditions (gamma, alpha, sham) were entered as separate binary predictors. It would be more appropriate and interpretable to model the tDCS condition as a single, three-level categorical factor, given that both sham and alpha serve as control conditions. A model structure including Inequality_Type x tDCS_conditions as regressors would allow for a more direct test of whether gamma stimulation differs from both sham and alpha stimulation, thereby strengthening the specificity and interpretability of the findings. It is essential that the effect of gamma frequencies is not only significant in contrast to sham but also significantly different from alpha, which needs to be reflected in the LMER model results by a significant interaction effect of Inequality_Type x tDCS_conditions.*

**Response:**

We thank the reviewer for raising this point. We fully agree that it is important to demonstrate that the effect of gamma stimulation is significant not only relative to sham but also relative to alpha.

However, given the model we already report, we are unsure whether the reviewer suggested alternative analyses to test for these effects, or whether our description of the analysis may have been unclear and may have led to a misunderstanding. In the lmer model specification we report in the current manuscript, we specified stimulation condition as a three-level categorical factor (gamma, alpha, sham). This is mathematically equivalent to entering it as two binary dummy variables (gamma vs. sham and gamma vs. alpha, as in our original model), since a factor with k levels is internally decomposed into k–1 dummy variables under the categorical factor coding in lmer (see the R documents of “*factor*”). Thus, our original modeling approach is structurally identical to the model suggested by the reviewer. The difference lies only in parameterization and coefficient labeling, not in the statistical test itself (see e.g., Bates et al., lme4 documentation).

Note that alternative model structures specifying the tACS predictor as a single factor would not by themselves allow testing whether gamma differs from alpha or sham - this would still require specific post hoc contrasts. Moreover, this model specification would impose a specific linear shape on the effect of Inequality_Type x tACS_conditions (as opposed to the two independent tests of gamma vs sham and gamma vs alpha as implemented now). We therefore think our current model is a better test of our questions and the questions asked by the reviewer: it provides the required tests without imposing any assumptions about the specific relation between the sizes of two effects, and it does not require additional post-hoc analyses. We hope the reviewer agrees with this line of reasoning, which we have now made more explicit in the text (*see below*) to make sure the reader understands the rationale for our statistical analysis approach.

New text on *pp. 32-33*:

“To examine the effects of tACS on altruistic behavior, we performed a logistic mixed-effects regression predicting trial-wise choices (altruistic = 1, selfish = 0) as a function of inequality context (DIS = 0, ADV = 1), stimulation condition, and their interaction. The stimulation condition was modeled as a categorical factor with three levels (gamma, alpha, sham). In the lme4 package for R, a categorical predictor with *k* levels is internally represented by *k–1* dummy variable under treatment (reference) coding, such that each estimated coefficient reflects the contrast between one active condition (i.e., gamma entrainment) and the reference condition (i.e., alpha or sham stimulation). This approach is mathematically equivalent to the conventional dummy coding representation of a three-level categorical factor in mixed-effects models (e.g., dummy variables: gamma entrainment (gamma = 1, sham or alpha = 0); sham stimulation (sham = 1, gamma or alpha = 0); and alpha entrainment (alpha = 1, sham or gamma = 0)). The difference lies only in parameter labeling, not in the estimated model structure or statistical tests. This approach has several statistical advantages. First, logistic mixed-effects regression accounts for random subject- and item-level variability, thereby improving statistical power and generalizability compared to subject-wise estimates followed by t-tests. Second, testing contrasts within the model avoids the problem of “double dipping” that would arise if one first extracted estimates and then performed secondary tests. Third, corrections for multiple comparisons are inherently controlled when specifying orthogonal contrasts in the mixed model framework.”

*(2) For a further understanding of how these three conditions (gamma, alpha, sham) are related to each other, a post-hoc pairwise comparison between the three tDCS levels (e.g., gamma vs. sham, gamma vs. alpha, alpha vs. sham) should be reported, applying appropriate corrections for multiple comparisons. Based on these regression models, as previously described by the authors, one-tailed/ two-tailed statistical tests can be calculated (e.g., by extracting individual estimates from these models).*

**Response:**

We thank the reviewer for this valuable suggestion. We agree that it is critical to clarify precisely how the pairwise comparisons between the three stimulation conditions (gamma, alpha, sham) were tested. In our revision, we now explicitly report the contrasts of interest.

Specifically, in the current linear mixed-effects model, the stimulation conditions were entered as categorical factor variable (mathematically equivalent to treatment coding with dummy variables). This parameterization allows us to test the contrasts of gamma vs. sham and gamma vs. alpha directly within the regression framework, while alpha vs. sham can be obtained as well. Thus, the LMM naturally provides all pairwise comparisons of interest without requiring separate ANOVAs and post-hoc t-tests.

Importantly, this approach has several statistical advantages. First, LMMs account for random subject- and item-level variability, thereby improving statistical power and generalizability compared to subject-wise estimates followed by t-tests. Second, testing contrasts within the model avoids the problem of “double dipping” that would arise if one first extracted estimates and then performed secondary tests. Third, corrections for multiple comparisons are inherently controlled when specifying orthogonal contrasts in the mixed model framework.

We have now made these points explicit in the Methods section (*pp. 32-33*, see the response to the above comment), and we report the pairwise comparisons (gamma vs. sham, gamma vs. alpha, alpha vs. sham) clearly, thereby ensuring full transparency of how the effects were tested. We believe this provides a clearer and statistically more principled solution than an ANOVA with post-hoc tests.

*(3) The results of the LMER models and post-hoc analyses should be presented in corresponding tables. It is not necessary to include model outputs with control variables such as discomfort in the tables; these can be briefly summarized in the Results section to demonstrate the robustness of the main findings.*

**Response:**

Thank you very much. We have followed the reviewer’s helpful recommendation (see updated Table 1 on *p. 45* in the main text). We have now moved the detailed LMER model outputs (including discomfort/current intensity) to the Supplementary Information.

*Limitations of tDCS Montage and Source Analysis:*

*(1) Based on the SimNIBS simulations, the frontal-parietal network that was targeted looks quite widespread. This should be described as a limitation in the discussion, as it reduces the focal specificity of the findings.*

**Response:**

We agree that the SimNIBS simulations indicate a relatively widespread stimulation pattern across the fronto-parietal network, which limits the focal specificity of our findings. We have now explicitly acknowledged this in the Discussion. At the same time, we note that our use of high-definition tACS represents one of the most focal non-invasive approaches currently available for modulating long-range connectivity in specific oscillatory frequency bands, and in particular for manipulating frequency-specific phase-coupling. Nevertheless, we recognize that future studies employing even more spatially precise methods (e.g., individualized current modeling, neuronavigation-guided montages, or combined TMS-EEG approaches) will be needed to further refine the causal mapping of subregions within the fronto-parietal circuitry.

We now critically discuss these issues in the manuscript (*p. 20*): “Our current field simulations indicated that the present montage produced relatively widespread current flow within the fronto-parietal network. This reduces the focal specificity of our conclusions, since the behavioral effects observed cannot be attributed to a narrowly defined subregion. Nevertheless, our use of high-definition (HD) tACS represents one of the most focal non-invasive approaches currently available for modulating long-range connectivity in specific oscillatory frequency bands, and in particular for manipulating frequency specific phase-coupling. Moreover, although the simulated electric field may have extended partially toward the DLPFC and other frontal areas, the field strength in these areas (approximately 0.0208–0.0330 V/m, Table S1) was markedly lower than that beneath the main electrodes (about 0.13–0.14 V/m), suggesting minimal direct modulation of DLPFC or other frontal regions activity. These findings suggest that although minor current spread reached adjacent frontal regions, the effective stimulation within these areas was considerably weaker than in the intended fronto-parietal target network associated with altruistic decision-making. Importantly, the stimulation parameters were identical across experimental conditions (i.e., both gamma and alpha active control), indicating that any potential off-target effects would not confound the frequency-specific nature of our results. Taken together, our results are best interpreted as evidence that HD-tACS modulated gamma-band synchronization at the network level of the fronto-parietal circuit, rather than as causal effects brought about by stimulation of a single cortical locus. Future studies building on our approach could improve spatial specificity by using individualized current modeling, neuronavigation-guided montages to target individual fMRI-defined areas, or combined TMS-EEG approaches to directly measure the neural consequences of modulating frequency-specific coherence in the fronto-parietal circuitry.”

*(2) The authors describe the tDCS montage based on the area where gamma rhythms during the altruistic choice process were recorded, building on their previous EEG results. Nonetheless, as EEG has low spatial resolution and signals can be recorded in one region while originating from another, it still remains essential to specify which exact areas within the frontal and parietal cortices were targeted by tDCS. It would be particularly informative to assess whether the stimulated regions align with areas previously implicated in prosocial and altruistic decision-making (i.e., those involved in weighing other-related concerns), using MNI coordinates reported in prior studies. Linking the present stimulation sites to such findings would help clarify the specific type of altruistic behavior influenced by brain stimulation. For example, it would be relevant to examine whether stimulation targets part of the ventromedial prefrontal cortex, which has been previously associated with promoting other-regarding preferences (Hutcherson et al., 2015). Such clarification would also strengthen the novelty of the findings by highlighting how tDCS, in contrast to previous EEG results, contributes to understanding the focal specificity of frontal brain regions involved in promoting altruistic choices.*

**Response:**

We thank the reviewer for this valuable suggestion. To clarify the specific cortical regions targeted by our tACS montage, we conducted additional analyses to map the simulated electric field distributions onto MNI coordinates corresponding to regions previously implicated in other-interest processing and altruistic decision-making (Hutcherson et al., 2015; Hu et al., 2017; Morishima et al., 2012). Specifically, we calculated the mean simulated electric field within 8-mm-radius spheres centered on MNI coordinates derived from (a) prior fMRI studies on altruistic and other-regarding choices, and (b) a Neurosynth meta-analysis using the term “social interaction” (see Table S1).

These analyses covered key frontal regions—including the ventromedial prefrontal cortex (VMPFC: 6, 38, 9; −4, 44, −8), dorsomedial prefrontal cortex (DMPFC: 2, 56, 16), anterior cingulate cortex (ACC: 9, 36, 3), middle cingulate cortex (MCC: 1, 9, 30; 2, 26, 42), dorsolateral prefrontal cortex (DLPFC: 51, 11, 22), and inferior frontal gyrus (IFG: 52, 30, 4)—as well as parietal regions such as the temporoparietal junction (TPJ: 52, −51, 27; 63, −42, 21) and inferior parietal lobule (IPL: 57, −37, 52).

Simulations (SimNIBS; stimulation intensity = 3.2 mA peak-to-peak) indicated that the electric field strength beneath the two main electrodes reached approximately 0.13–0.14 V/m—substantially higher than most other cortical regions examined. Notably, the central parietal electrode was positioned directly over the right IPL (57, −37, 52, bold text in Table S1)—a region implicated in other-interest processing (Hu et al., 2017) where the mean field strength reached 0.113 V/m (max = 0.145 V/m). Two nearby parietal sites, corresponding to the right TPJ (63, −42, 21; 51, −51, 27; bold text in Table X), also exhibited moderate stimulation levels (mean = 0.075 and 0.072 V/m, respectively), and these sites are consistent with areas found previously to correlate in their activity with other-regarding preferences (Hutcherson et al., 2015; Morishima et al., 2012).

In contrast, most frontal regions received less than half of the field strength observed beneath the central electrodes. Only the left superior frontal gyrus (SFG; −22, 19, 60, bold text in Table S1) exhibited a moderate field (mean = 0.077 V/m), consistent with its reported role in other-interest processing (Hutcherson et al., 2015).

Overall, these analyses demonstrate that the stimulated regions overlapped with key nodes previously implicated by fMRI studies in other-regarding and altruistic decision-making networks, thereby reinforcing the functional relevance of our tACS montage. In the revised manuscript (*see below*), we have clarified the stimulation intensity for simulation analyses (3.2 mA peak-to-peak), reported the corresponding simulated field strengths (~0.13–0.14 V/m), and added Table S1 below in the supplementary materials, which lists mean field strengths across all regions of interest. These additions strengthen the anatomical and functional specificity of our stimulation protocol and clarify its relation to established altruistic neural networks.

New text on *pp. 9-10*:

“To better understand which cortical regions were targeted by our tACS montage, we simulated electric field distributions and mapped them onto MNI coordinates corresponding to regions previously implicated (by fMRI studies) in altruistic and other-regarding decision-making (Hu et al., 2017; Hutcherson et al., 2015; Knoch et al., 2006; Morishima et al., 2012; Sanfey et al., 2003), or found to be involved in “social interaction” based on Neurosynth meta-analysis (Table S1).

Simulations were performed using SimNIBS with 3.2 mA peak-to-peak stimulation intensity, see Methods section for more details (Thielscher et al., 2015; Van Hoornweder et al., 2024). The results indicated that the electric field strength beneath the two main electrodes reached approximately 0.13–0.14 V/m, which was substantially higher than in most other cortical regions examined. Notably, the central parietal electrode was positioned directly over the right IPL (57, −37, 52; bold in Table S1), a region previously implicated in other-interest processing (Hu et al., 2017), where the mean field strength reached 0.113 V/m (max = 0.145 V/m). Two nearby parietal sites corresponding to the right TPJ (63, −42, 21; 51, −51, 27; bold in Table S1) also exhibited moderate stimulation levels (mean = 0.075 and 0.072 V/m, respectively), consistent with prior findings that link TPJ activity to other-regarding preferences (Hutcherson et al., 2015; Morishima et al., 2012).

By contrast, most frontal regions received less than half of the field strength observed beneath the central electrodes. Only the left superior frontal gyrus (SFG; −22, 19, 60; bold in Table S1) exhibited a moderate electric field (mean = 0.077 V/m), aligning with prior reports of its role in other-interest processing (Hutcherson et al., 2015).

Overall, these additional analyses demonstrate that the stimulated regions overlapped with key nodes previously implicated by fMRI studies in other-regarding and altruistic decision-making networks, thereby reinforcing the functional relevance of our tACS montage. The present findings extend beyond our previous EEG-based results, by highlighting the anatomical and functional characteristics of the targeted brain regions.”

New text on *pp. 31-32*:

“*Electric field simulation*

To clarify the specific cortical regions targeted by our tACS montage, we conducted additional analyses to map the simulated electric field distributions onto Montreal Neurological Institute (MNI) coordinates corresponding to brain regions previously implicated in other-interest processing and altruistic decision-making (Hu et al., 2017; Hutcherson et al., 2015; Knoch et al., 2006; Morishima et al., 2012; Sanfey et al., 2003) or in “social interaction” based on Neurosynth meta-analysis. Specifically, we calculated the mean simulated electric field within 8-mm-radius spheres centered on MNI coordinates derived from (a) prior fMRI studies on altruistic and other-regarding choices, and (b) a Neurosynth meta-analysis using the term “social interaction” (Table S1) with SimNIBS (Thielscher et al., 2015; Van Hoornweder et al., 2024).

These analyses covered key frontal regions—including the ventromedial prefrontal cortex (VMPFC: 6, 38, 9; −4, 44, −8), dorsomedial prefrontal cortex (DMPFC: 2, 56, 16), anterior cingulate cortex (ACC: 9, 36, 3), middle cingulate cortex (MCC: 1, 9, 30; 2, 26, 42), dorsolateral prefrontal cortex (DLPFC: 51, 11, 22), and inferior frontal gyrus (IFG: 52, 30, 4)—as well as parietal regions such as the temporoparietal junction (TPJ: 52, −51, 27; 63, −42, 21) and inferior parietal lobule (IPL: 57, −37, 52).”

Table S1. Simulated electric field intensities at regions of interest derived from prior studies on other-regarding preferences and altruistic decision-making.

| Regions | Peak coordinates | | | Contrasts/Key term | Mean intensity in a sphere of 8 mm radius (V/m) | References |
| --- | --- | --- | --- | --- | --- | --- |
|  | x | y | z |  |  |  |
| **Prefrontal cortice** |  | | |  |  |  |
| R ACC | 9 | 36 | 3 | Other-interest | 0.0162 | Hutcherson et al., 2015 |
| R MCC | 1 | 9 | 30 | Other-interest $\cap$ Self-interest | 0.0410 |  |
| **L SFG** | **-22** | **19** | **60** |  | **0.0770** |  |
| R VMPFC | 6 | 38 | 9 |  | 0.0185 |  |
| R DLPFC | 51 | 11 | 22 |  | 0.0309 | Hu et al., 2017 |
| L DLPFC | -40 | 38 | 23 | Unfair > Fair | 0.0230 | Knoch et al., 2006; Sanfey et a., 2003 |
| R DLPFC | 40 | 37 | 22 |  | 0.0208 |  |
| R ACC | 4 | 19 | 39 |  | 0.0426 | Sanfey et al., 2003 |
| L ACC | -9 | 26 | 28 |  | 0.0320 |  |
| R MFG | 34 | 23 | 35 |  | 0.0370 |  |
| L MFG | -39 | 21 | 41 |  | 0.0538 |  |
| L VMPFC | -4 | 44 | -8 | Social interaction | 0.0104 | Meta-analyses in Neurosynth |
| R DMPFC | 2 | 56 | 16 |  | 0.0137 |  |
| R MCC | 2 | 26 | 42 |  | 0.0427 |  |
| L DLPFC | -44 | 18 | 24 |  | 0.0256 |  |
| R DLPFC | 50 | 10 | 22 |  | 0.0330 |  |
| L IFG | -48 | 30 | -8 |  | 0.0088 |  |
| R IFG | 52 | 30 | 4 |  | 0.0147 |  |
| **Parietal cortice** |  |  |  |  |  |  |
| **R TPJ** | **63** | **-42** | **21** | Altruistic preference/decision | **0.0747** | Morishima, 2012 |
|  | 60 | -44 | 18 |  | 0.0598 |  |
|  | **51** | **-51** | **27** | Other-interest > Self-interest | **0.0715** | Hutcherson et al., 2015 |
|  | 39 | -63 | 21 | Other-interest | 0.0440 |  |
| **R IPL** | **57** | **-37** | **52** |  | **0.1132** | Hu et al., 2017 |
| R TPJ | 58 | -42 | 12 | Social interaction | 0.0459 | Meta-analyses in Neurosynth |
| L TPJ | -50 | -60 | 24 |  | 0.0182 |  |

ACC, Anterior cingulate cortex; MCC, Middle cingulate cortex; SFG, Superior frontal gyrus; VMPFC, Ventromedial prefrontal cortex; DLPFC, Dorsolateral prefrontal cortex; DMPFC, Dorsomedial prefreont cortex; IFG, Inferior frontal gyrus; MFG, Middle frontal gyrus; TPJ, temporoparietal junction; IPL, inferior parietal lobe; R, Right; L, Left.

*(3) The SimNIBS simulation suggests that tDCS stimulation might have targeted adjacent brain regions, such as the Dorsolateral Prefrontal Cortex (DLPFC), in addition to the fronto-parietal network linked to altruistic behavior. Given the ongoing debate about the DLPFC's role in social decision-making, particularly whether it promotes prosocial or selfish tendencies in social decision-making (e.g., presence of punishment; Knoch et al., 2006; Buckholtz et al., 2013) with current evidence suggesting that rDLPFC does not promote prosocial giving (Christian et al., 2022), I would recommend calculating an additional source localization analysis to assess whether tDCS stimulation targeted adjacent areas such as the DLPFC, and if necessary, discuss this shortly as a further limitation.*

**Response:**

We agree that it is important to clarify whether our tACS stimulation extended to the DLPFC or other adjacent frontal regions. However, we would like to note that the source localization analyses mentioned by the reviewer are typically applied to EEG data, rather than to electric field simulations. To address the reviewer’s question, we evaluated the electric field strength at MNI coordinates corresponding to key frontal regions reported in prior literature—specifically, the VMPFC, DMPFC, ACC, MCC, DLPFC, IFG, and SFG (see Table S1).

Our simulations indicated that the average field strength in the DLPFC ranged between 0.0208 and 0.0330 V/m, and in most other frontal regions between 0.0088 and 0.0538 V/m. This is substantially lower than the strength observed beneath the main frontal-parietal electrodes (approximately 0.13–0.14 V/m). Only the left superior frontal gyrus (SFG; −22, 19, 60, bold text in Table S1) exhibited a moderate field (mean = 0.077 V/m), but has previously also been reported to be involvemed in other-interest processing (Hutcherson et al., 2015).

These findings suggest that although minor current spread reached adjacent frontal regions, the effective stimulation within these areas was considerably weaker than in the intended fronto-parietal target network associated with altruistic decision-making. Importantly, the stimulation parameters were identical across experimental conditions (i.e., both gamma and alpha active control), indicating that any potential off-target effects would not confound the frequency-specific nature of our results.

We now explicitly discuss this issue in the Discussion section of the revised manuscript (*p. 20*): “Our current field simulations indicated that the present montage produced relatively widespread current flow within the fronto-parietal network. This reduces the focal specificity of our conclusions, since the behavioral effects observed cannot be attributed to a narrowly defined subregion. Nevertheless, our use of high-definition (HD) tACS represents one of the most focal non-invasive approaches currently available for modulating long-range connectivity in specific oscillatory frequency bands, and in particular for manipulating frequency specific phase-coupling. Moreover, although the simulated electric field may have extended partially toward the DLPFC and other frontal areas, the field strength in these areas (approximately 0.0208–0.0330 V/m, Table S1) was markedly lower than that beneath the main electrodes (about 0.13–0.14 V/m), suggesting minimal direct modulation of DLPFC or other frontal regions activity. These findings suggest that although minor current spread reached adjacent frontal regions, the effective stimulation within these areas was considerably weaker than in the intended fronto-parietal target network associated with altruistic decision-making. Importantly, the stimulation parameters were identical across experimental conditions (i.e., both gamma and alpha active control), indicating that any potential off-target effects would not confound the frequency-specific nature of our results. Taken together, our results are best interpreted as evidence that HD-tACS modulated gamma-band synchronization at the *network level* of the fronto-parietal circuit, rather than as causal effects brought about by stimulation of a single cortical locus. Future studies building on our approach could improve spatial specificity by using individualized current modeling, neuronavigation-guided montages to target individual fMRI-defined areas, or combined TMS-EEG approaches to directly measure the neural consequences of modulating frequency-specific coherence in the fronto-parietal circuitry.”

*Minor Revisions*

*(1) The manuscript repeatedly refers to potential clinical implications of modulating frontoparietal connectivity for promoting altruistic behavior, particularly for autism or alexithymia. For example, the statement, "Experimentally clarifying the causal role of frontoparietal connectivity for altruism may be a necessary step towards developing and/or improving intervention approaches to facilitate altruism, e.g., in people affected by social apathy and other related psychiatric and neurological disorders," is overly general and somewhat outdated, especially regarding individuals with autism. The term "social apathy" lacks clinical specificity and requires a more precise description based on current clinical literature. Moreover, recent research (e.g., Okuzumi, 2024) challenges the assumption that individuals with autism are inherently less altruistic than neurotypical individuals. Instead, difficulties in interpreting and responding to others' social cues—rather than a reduced capacity for altruism itself—are more accurate characterizations of social challenges in autism and alexithymia. Thus, I recommend that the authors specify which components of altruistic behavior may be atypical in these populations (e.g., perspective-taking) and clarify how tDCS might modulate these particular processes. More importantly, I suggest weakening or removing claims about clinical relevance or therapeutic applications, as enhancing altruism per se may be less meaningful than addressing the underlying mechanisms of social understanding that influence prosocial behavior.*

**Response:**

We appreciate the reviewer’s thoughtful comments regarding the interpretation of our findings in relation to clinical populations. We agree that our initial statement was too general and may not accurately reflect current clinical perspectives. Specifically, it is important to clarify which aspects of altruistic behavior may be atypical in different populations. For example, in psychopathy, atypical tendencies comprise reduced empathic concern and emotional responsiveness, which in turn can constrain prosocial or altruistic behaviors (Blair, 2008; Marsh, 2018). By contrast, in autism spectrum conditions, recent studies suggest that individuals are not inherently less altruistic; rather, difficulties often arise from challenges in mentalizing and interpreting social cues (Bölte, 2025; Livingston et al., 2019). Similarly, in alexithymia, deficits in recognizing and labeling one’s own and others’ emotional states can interfere with empathic processes that support altruism (Bird & Viding, 2014).

In light of these distinctions, our study does not allow strong conclusions about specific clinical applications. While our stimulation approach may modulate frontoparietal connectivity, it remains unclear whether this would selectively influence perspective-taking, emotional resonance, or other processes underpinning altruism. Therefore, rather than suggesting direct therapeutic applications, we propose that future work should investigate whether tACS differentially modulates specific components of altruistic behavior across populations. Such studies would provide more converging evidence on the underlying mechanisms, which could, in the longer term, inform clinical translation in a more targeted way. We hope the reviewer agrees that such a general discussion (*see below*) of the potential implications of our findings is warranted.

New text on *p. 24*:

“Our findings could have clinical implications, but these at present have to remain somewhat speculative: Altruistic behavior is not uniformly reduced across clinical populations; rather specific components of this behavior may be atypical in different disorders. For instance, in psychopathy, atypical tendencies comprise reduced empathic concern and emotional responsiveness, which in turn can constrain prosocial or altruistic behaviors (Blair, 2008; Marsh, 2018). By contrast, in autism spectrum conditions, recent studies suggest that individuals are not inherently less altruistic; rather, difficulties often arise from challenges in mentalizing and interpreting social cues (Bölte, 2025; Livingston et al., 2019). Similarly, in alexithymia, deficits in recognizing and labeling one’s own and others’ emotional states can interfere with empathic processes that support altruism (Bird & Viding, 2014). In light of these distinctions, our study does not yet allow strong conclusions about specific clinical applications. While our stimulation approach may modulate frontoparietal connectivity, it remains unclear whether this would selectively influence perspective-taking, emotional resonance, or other processes underpinning altruism. We therefore propose that future work should investigate whether tACS differentially modulates distinct components of altruistic behavior across different populations. Such studies would provide evidence for more specific cognitive or affective mechanisms affected by the stimulation, which would inform clinical translation in a more targeted way.”

*(2) In the introduction, the authors describe the role of frontal brain areas in prosocial decision-making in broad terms, without specifying the precise role of distinct frontal brain regions in self- versus other-oriented behavior. This weakens the theoretical clarity of the manuscript. Given that the role of the prefrontal cortex for social decision-making is highly context-dependent, it is important that the authors describe this in more detail which distinct parts of the prefrontal cortex are involved or causally relevant in promoting selfish or other-related interests.*

**Response:**

We thank the reviewer for pointing out the need for greater theoretical clarity regarding the role of distinct prefrontal regions in self- versus other-oriented behavior. We have now expanded the Introduction to provide a more differentiated overview of the prefrontal cortex in prosocial decision-making. Specifically, we highlight that the ventromedial prefrontal cortex (VMPFC) has been consistently implicated in representing the subjective value of prosocial options and integrating other-regarding preferences (Hare et al., 2010; Sul et al., 2015). By contrast, the dorsolateral prefrontal cortex (DLPFC) is more strongly associated with implementing control processes that may either promote self-interest or support norm compliance and fairness depending on contextual demands (Baumgartner et al., 2011; Buckholtz et al., 2015). Additionally, the anterior cingulate cortex (ACC) has been shown to monitor conflicts between self- and other-regarding motives, and to track the cost of prosocial acts (Apps & Ramnani, 2014). While fMRI studies allow for such region-specific insights, EEG provides more limited spatial resolution. Nevertheless, our previous EEG results suggested that it is central prefrontal activity that is particularly involved in weighing other-related interests, which is unlikely to primarily reflect DLPFC functions. Together, this extension provides a clearer theoretical framework for interpreting our results.

We now outline this in the manuscript (*pp. 3-4*): “Importantly, the evidence suggests that distinct regions of the prefrontal cortex may contribute differently to self- versus other-oriented behavior: While the ventromedial prefrontal cortex (VMPFC) has been consistently implicated in representing the subjective value of prosocial options and integrating other-regarding preferences (Hare et al., 2010; Sul et al., 2015),the dorsolateral prefrontal cortex (DLPFC) has been more strongly associated with implementing control processes that may either promote self-interest or support norm compliance and fairness depending on contextual demands (Baumgartner et al., 2011; Buckholtz et al., 2015). As a third region, the anterior cingulate cortex (ACC) has been shown to monitor conflicts between self- and other-regarding motives, and to track the cost of prosocial acts (Apps & Ramnani, 2014). Moreover, when people are taking altruistic choices, neural responses in medial prefrontal cortex and TPJ are stronger than when they are taking selfish choices (Hutcherson et al., 2015; Morishima et al., 2012). While fMRI studies allow for such region-specific insights, EEG provides more limited spatial resolution. Nevertheless, our previous EEG results suggested that it is central prefrontal and parietal activity that is particularly involved in weighing other-related interests, which is unlikely to primarily reflect lateral DLPFC functions (Hu et al., 2022). Together, these findings highlight that altruistic decision-making cannot be attributed to a single frontal locus but instead relies on partially dissociable and interacting prefrontal processes, which form functional networks with parietal regions (Hare et al., 2010; Hu et al., 2017; Hutcherson et al., 2015).”

In closing, we would like to thank both reviewers for their insightful and helpful comments, which have led us to implement changes that have improved our manuscript. I hope you agree that it is now ready for publication.
